# Probiotics as a Complementary Medicine in Neurologic Disorders

**DOI:** 10.1002/hsr2.71422

**Published:** 2025-10-28

**Authors:** Mahla Jafari, Morteza Alipour, Sara Zamani, Aryan Mohtasham Amiri, Parsin Pourabbas, Meysam Hasannejad‐Bibalan

**Affiliations:** ^1^ School of Medicine Guilan University of Medical Sciences Rasht Iran; ^2^ Biomedical Physics Group, Physics School University of Hamburg Hamburg Germany; ^3^ Student Research Committee, School of Medicine Guilan University of Medical Sciences Rasht Iran; ^4^ Department of Microbiology, School of Medicine Guilan University of Medical Sciences Rasht Iran

**Keywords:** Complementary and Integrative Medicine, gut–brain axis, microbiota–gut–brain interaction, neurological disorders, probiotics

## Abstract

**Background and Aims:**

Today, neurological and neuropsychiatric disorders, including depression, anxiety, Parkinson's disease (PD), Alzheimer's disease (AS), autism spectrum disorder (ASD), and multiple sclerosis (MS), contribute significantly to global disability and healthcare burden. Most current treatment options only provide symptomatic relief and are limited by challenges such as drug resistance, systemic side effects, and poor blood‐brain barrier permeability. The growing interest in the gut‐brain axis has encouraged exploration of the gut microbiota as a potential therapeutic target. Probiotics—live microorganisms that may confer health benefits to the host—have been proposed to modulate the gut‐brain axis through immune, metabolic, and neurochemical pathways.

**Methods:**

In this narrative review, a targeted search was performed across multiple databases to identify relevant articles, from which the key relationships and strategies were extracted. Then, we represented the findings to provide a comprehensive overview of the topic and highlight emerging trends and gaps in the literature.

**Results:**

Emerging preclinical and clinical studies suggest that specific probiotic strains can improve neurological symptoms by reducing neuroinflammation, supporting gut barrier integrity, and influencing neurotransmitter production. However, findings remain heterogeneous due to strain specificity, individual microbiome diversity, and methodological differences across studies. Preclinical and clinical studies suggest that specific probiotic strains can improve neurological symptoms by reducing neuroinflammation, enhancing gut barrier integrity, and influencing neurotransmitter production. Evidence supports their potential as adjunctive treatments for major neurological and neuropsychiatric disorders, including depression, anxiety, ASD, PD, AD, and MS, particularly in patients with gut dysbiosis or gastrointestinal comorbidities. However, findings remain heterogeneous due to strain specificity, individual microbiome variability, and methodological differences across studies.

**Conclusion:**

This brief review summarizes the current evidence on the use of probiotics in neurological disorders, discusses potential mechanisms of action, and highlights safety considerations and limitations. Future directions include personalized probiotic therapies, large‐scale randomized controlled trials, and integration with conventional neurological therapies. Overall, probiotics could be a low‐risk, complementary option in the evolving field of neurotherapy, but more rigorous evidence is needed before definitive clinical recommendations can be made.

## 1 Introduction

Neurological disorders are among the leading causes of premature mortality and long‐term disability worldwide [1]. In 2021, these conditions accounted for approximately 443 million disability‐adjusted life years (DALYs), affecting more than 3.4 billion individuals, nearly half of the global population. The burden of neurological diseases continues to rise, largely due to population aging, with Asia bearing a particularly high impact owing to its large elderly population and significant socioeconomic disparities. Countries such as China and India contribute substantially to global neurological morbidity, making this region central to understanding broader epidemiological trends [2].

Despite advancements in neuroscience and pharmacology, existing therapies for central nervous system (CNS) disorders largely provide symptomatic relief rather than disease modification or reversal. Conditions such as amyotrophic lateral sclerosis (ALS) and multiple sclerosis (MS) remain poorly responsive to current medications [3, 4, 5]. Moreover, long‐term pharmacological treatments often result in limited improvements in quality of life and are frequently associated with adverse effects. Drug delivery challenges, particularly the restrictive nature of the blood brain barrier (BBB), further limit the efficacy of many therapeutic agents, as most molecules fail to reach therapeutic concentrations within neural tissues [3]. This therapeutic gap highlights the urgent need for novel, safe, and effective strategies that target disease mechanisms beyond conventional pharmacology.

The gut–brain axis (GBA), a bidirectional communication network linking the gastrointestinal (GI) tract and central nervous system, has gained substantial attention for its role in the pathogenesis of neurological and neuropsychiatric disorders. The gut microbiota influences brain function through immune, metabolic, and neural pathways, modulating key neurotransmitters such as serotonin, dopamine, and γ‐aminobutyric acid (GABA) [6]. Dysbiosis, or disruption of the gut microbial ecosystem, has been implicated in disorders including autism spectrum disorder (ASD), depression, Alzheimer's disease, and Parkinson's disease [7]. However, while the link between dysbiosis and neurological disease has been increasingly recognized, there remains a lack of consolidated evidence on how microbiota‐targeted interventions, particularly probiotics, can be leveraged as adjunctive therapies.

Emerging evidence points to the therapeutic potential of psychobiotics, probiotic strains that influence mental health, through their ability to produce neuroactive metabolites and modulate microbiota composition [8]. Studies illustrated the important role of genetics in susceptibility of individuals to neurological disease [9, 10, 11, 12]. Advances in omics technologies have further deepened our understanding of gut microbial diversity, identifying potential biomarkers and therapeutic targets for neurological disease prevention and treatment. Nevertheless, findings across studies are heterogeneous, and the clinical applicability of probiotics in neurological disorders remains underexplored. Addressing this gap is essential to determine whether probiotics can move from experimental promise to meaningful clinical utility.

This mini‐review aims to synthesize current evidence regarding the role of probiotics as complementary therapies in neurological disorders. It outlines the mechanisms through which probiotics modulate the gut–brain axis, reviews relevant clinical and preclinical findings, and discusses safety considerations and future research directions. By explicitly focusing on probiotics within the broader gut–brain axis framework, this review seeks to clarify their potential role as low‐risk, adjunctive interventions for improving neurological health and patient outcomes.

## 2 Literature Search Strategy

To ensure transparency, a targeted narrative search of the scientific literature was conducted to identify relevant studies on probiotics and neurological disorders. Searches were conducted in PubMed/MEDLINE, Scopus, Web of Science, Embase, and the Cochrane Library up to August 31, 2025. The search terms included keywords and combinations of the terms: “probiotic” or “psychobiotic*“ or “synbiotic*“ and “depression” or “anxiety” or “Alzheimer's” or “dementia” or “Parkinson's” or “autism” or “ASD” or “MS”.

The search primarily considered selected preclinical studies (animal and laboratory) for mechanistic insights and human clinical trials where available. However, given the limited number of human clinical studies, preclinical evidence was emphasized. To clarify the scope, studies focusing on disorders such as amyotrophic lateral sclerosis (ALS), Huntington's disease, epilepsy, and traumatic brain injury were excluded due to very limited or heterogeneous human clinical data. Only English‐language publications were included in this review, which may introduce language bias.

## 3 Mechanisms of Probiotic Action in Neurology

### 3.1 Modulation of the Gut‐Brain Axis

**Figure 1:**
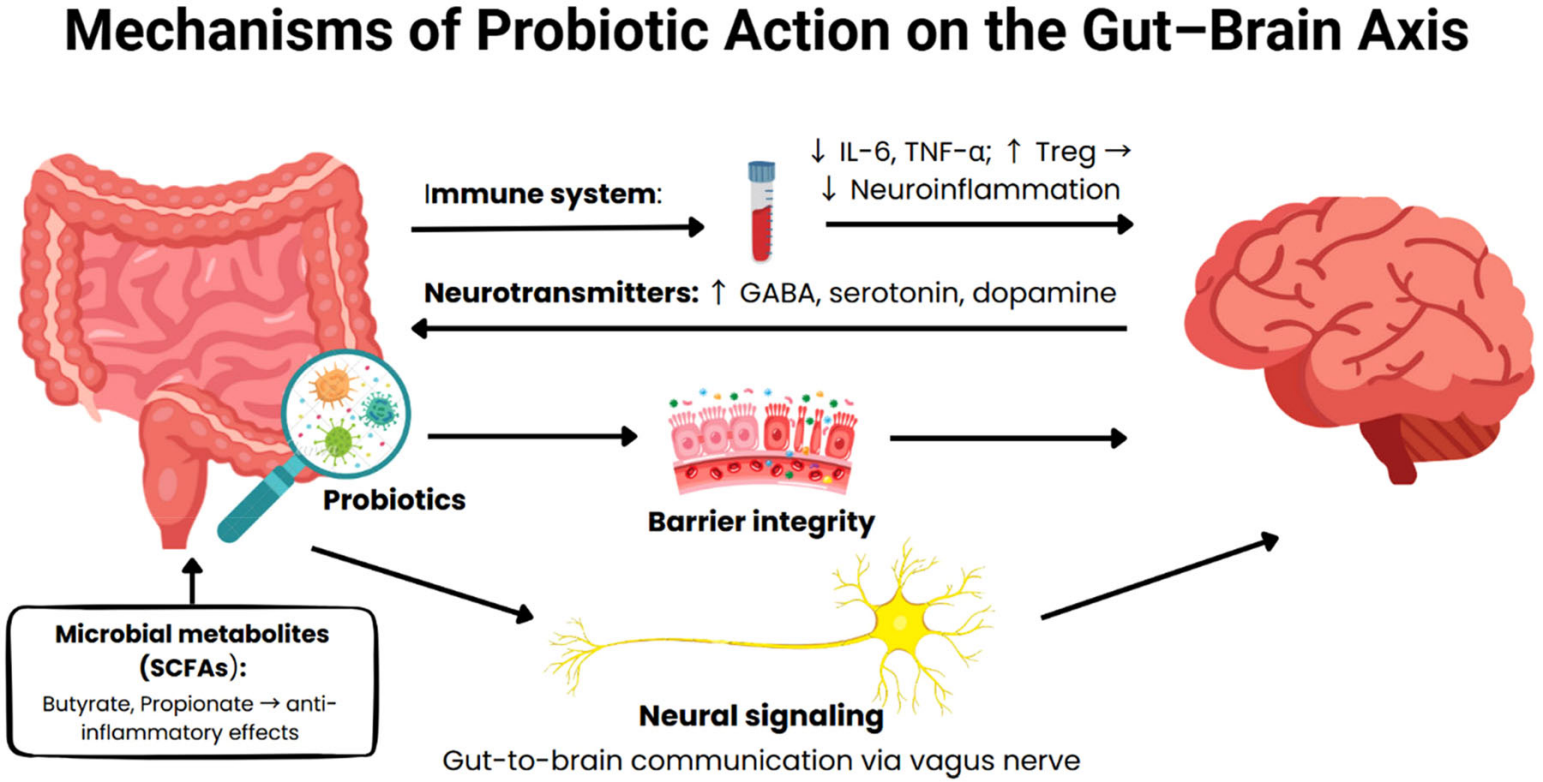
Mechanisms of probiotic action on the gut–brain axis.

Probiotics have emerged as promising adjunctive therapies for neurological disorders, primarily due to their modulatory effects on the GBA [13]. The GBA is a bidirectional communication network that links the enteric and central nervous systems through neural, immune, endocrine, and metabolic pathways [14]. Probiotics exert their influence via four principal mechanisms: modulation of the immune system, neurochemical signaling, maintenance of intestinal barrier integrity, and facilitation of gut–brain communication [15, 16, 17]. Probiotics influence the GBA by signaling through the vagus nerve, modulating the hypothalamic–pituitary–adrenal (HPA) axis, and generating microbial metabolites. Strains such as Lactobacillus plantarum and Streptococcus thermophilus, found in formulations like Bactolac, have been shown to activate vagal afferents and affect hippocampal receptors involved in mood regulation [18]. The vagus nerve detects microbial signals through gut chemoreceptors, while enteroendocrine cells establish direct connections with vagal fibers to facilitate gut‐to‐brain communication [19]. Probiotic administration can help restore GBA functionality that is often disrupted by dysbiosis or chronic stress [20] (Figure 1).

### 3.2 Immune Modulation and Reduction of Neuroinflammation

Probiotics contribute to immune homeostasis by enhancing regulatory T‐cell activity and reducing pro‐inflammatory cytokines such as IL‐6 and TNF‐α. They also promote the production of short‐chain fatty acids (SCFAs), notably butyrate, which mitigate inflammation by inhibiting histone deacetylases [21]. Moreover, probiotics help reinforce both the intestinal epithelial barrier and the BBB, thereby limiting peripheral immune activation that can impact the brain [22]. Through modulation of the kynurenine pathway, probiotics may promote the synthesis of neuroprotective kynurenic acid (KYNA) over neurotoxic quinolinic acid (QUIN) [15]. Clinical studies support these immunomodulatory effects; for instance, a multi‐strain probiotic formulation significantly reduced circulating IL‐6 and TNF‐α levels in patients with Parkinson's disease [23], suggesting a decrease in neuroinflammation relevant to conditions such as depression, Alzheimer's disease, Parkinson's disease, MS, and ASD [24].

### 3.3 Production of Neuroactive Compounds

Several probiotic strains are capable of producing or modulating key neurotransmitters, including GABA, dopamine, and glutamate [25]. *Bifidobacterium dentium* has been shown to synthesize GABA and increase tyrosine concentrations in both the colon and brain of mono‐associated mice [26]. Probiotic formulations like Bactolac have demonstrated the capacity to enhance the expression of serotonergic and dopaminergic receptors in the hippocampus [18]. In zebrafish, administration of *Lactobacillus rhamnosus* altered the expression of genes related to serotonin signaling and increased the relative abundance of Firmicutes, underscoring the microbiota's influence on neurochemical pathways [27].

### 3.4 Impact on Intestinal Permeability and Systemic Inflammation

Probiotics also play a critical role in maintaining intestinal barrier integrity by strengthening tight junctions and preventing the translocation of endotoxins, a process closely linked to neuroinflammation [28]. They help sustain mucosal integrity and reduce the permeability that allows pro‐inflammatory molecules to enter circulation [29]. *Lacticaseibacillus rhamnosus GG* has been shown to reinforce mucosal defenses and lower systemic cytokine levels associated with neurodegenerative diseases [21]. Bactolac has demonstrated protective effects on intestinal architecture under stress conditions, further emphasizing its potential therapeutic value [15, 16, 17, 18]. These mechanisms are interconnected: a robust intestinal barrier influences immune function, which in turn affects neurotransmission and overall brain health. However, continued clinical research is essential to elucidate strain‐specific effects and validate the therapeutic relevance of probiotics in neurological contexts.

### 3.5 Strain‐Specific vs Class‐Level Effects

Clinical evidence suggests that the efficacy of probiotics is primarily dependent on the “exact strain” and the “target disease”; therefore, pooling heterogeneous results and interpreting them at the class level can be misleading [30]. In the Alzheimer's triple‐arm trial, intervention with two distinct strains, Lactobacillus rhamnosus HA‐114 and Bifidobacterium longum R0175, produced distinct patterns in plasma free amino acids and pathways involved, highlighting the “strain‐specificity” of the effect even in a single disorder [31].

**Figure 2:**
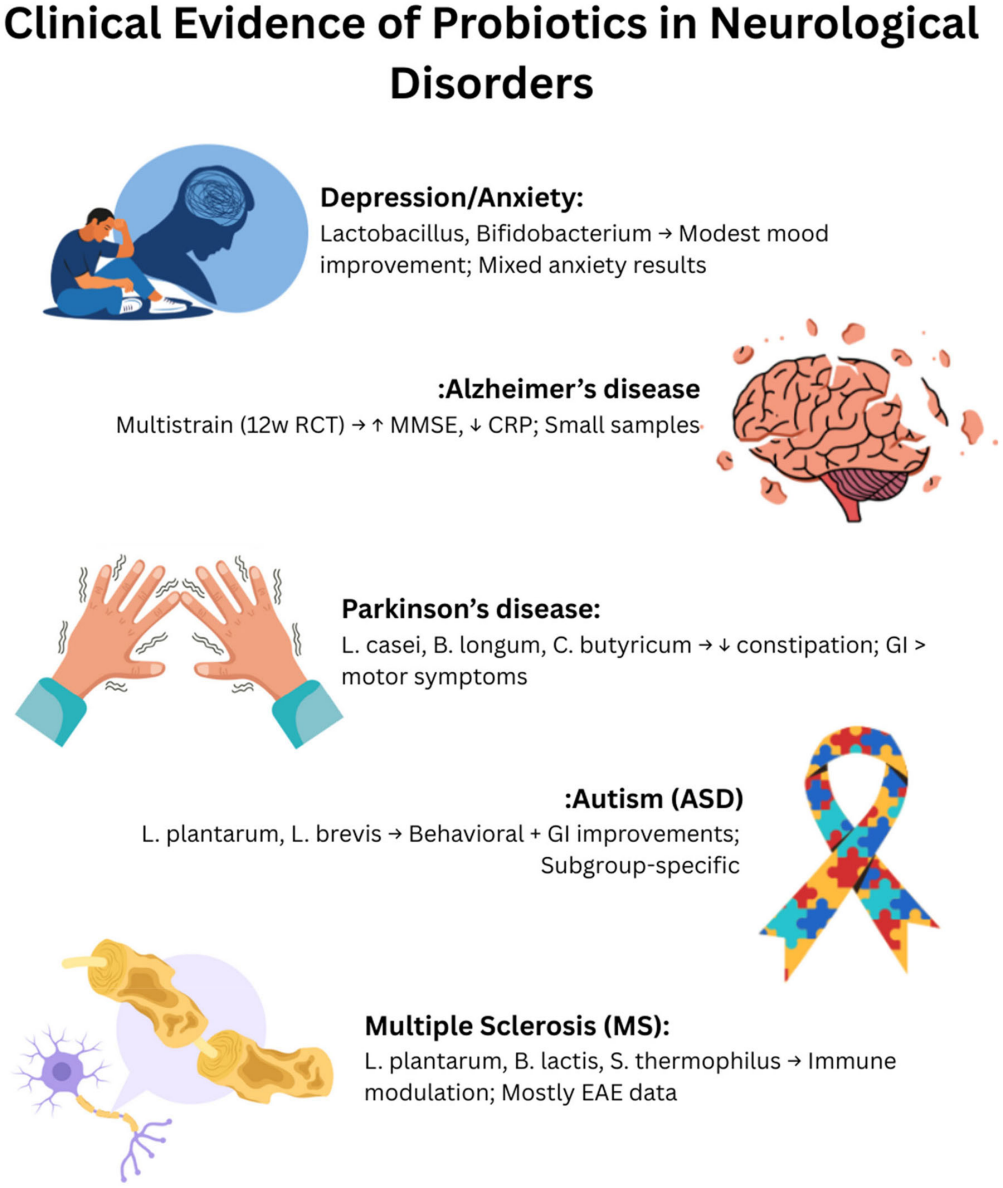
Clinical evidence of probiotics in neurological disorders.

However, “class/genus‐level” evidence has also been reported: in healthy elderly subjects, a multi‐strain formulation of common Lactobacillus/Bifidobacterium genera showed significant improvements in cognition and emotional symptoms (BDI/STAI); a result that is interpreted at the formulation/genus level, not a single strain [32]. Also, in major depression, increased abundance of the genus Lactobacillus in the probiotic group was associated with reduced HAM‐D/BDI scores, although data were reported at the “genus” level [33]. Overall, distinguishing “strain‐specific” from “class/genus” outcomes is essential to standardize strain selection, dosage, and formulation and avoid arbitrary pooling; it is recommended that clinical inferences be based primarily on the specific strain, and that class‐level evidence be used only contextually and with caution [30, 31, 32, 33] (Figure 2).

### 3.6 Probiotics Versus Prebiotics and FMT

Probiotics, prebiotics, and fecal microbiota transplantation (FMT) are among the approaches that have been proposed to modulate the gut microbiota and affect mental and neurological health. Probiotics are live microorganisms that help improve brain and nervous system function by producing beneficial metabolites such as SCFA and regulating immune responses [34]. Despite these positive effects, probiotics face practical limitations in drug delivery, particularly due to problems with persistence in the gut and limited ability to achieve specific therapeutic targets. For this reason, methods to improve their stability and more precise targeting have been proposed [35].

Prebiotics, which are non‐digestible substances, stimulate the growth of beneficial bacteria in the gut. These substances can also have positive effects on gut and overall health, similar to probiotics, but their effects may vary depending on the host's microbiota composition and may not be effective in individuals with specific microbial disorders [34, 35, 36, 37]. FMT, which involves the transfer of healthy microbiota from a donor to a recipient, has been proposed as a promising approach to treat neurological diseases such as Alzheimer's and Parkinson's. However, this approach faces challenges such as heterogeneity in microbial uptake, limited scalability, a lack of standardized protocols, and the need for careful screening of donors to prevent the transfer of pathogens and resistance genes [34, 35].

Overall, the available evidence suggests that all three of these approaches can improve mental and cognitive health through their metabolic and immune effects. Probiotics and prebiotics are more targeted tools with subtler effects, while FMT is capable of completely reorganizing the gut microbiota. In general, probiotics can be said to provide a more defined modulation and are safer, although their effects are usually less intense and dependent on specific strains. More research is needed to better understand the exact mechanisms of these effects (Table 1).

**Table 1:** Probiotics, prebiotics, and fecal microbiota transplantation (FMT) [34, 35, 37].

Dimension	Probiotics	Prebiotics	Fecal microbiota transplantation
**What it is**	Live microorganisms administered to confer a health benefit.	Non‐digestible substrates that selectively stimulate beneficial gut taxa.	Transfer of a complete, healthy donor microbiota to a recipient.
**Key gut–brain mechanisms**	Immune modulation; production of neuroactive/immune metabolites (e.g., SCFAs); effects on neurotransmission/HPA axis.	Selectively increase beneficial SCFA‐producing microbes; downstream immune–metabolic effects on the gut–brain axis.	Community‐level reconstitution; broad shifts in composition/metabolites with neuro‐immune consequences.
**Evidence in neuro/mental health**	Clinical signals across neurodegenerative/psychiatric contexts; effects are strain‐/disease‐specific and heterogeneous.	Supportive signals (often as defined fibers/substrates) with variability across hosts and studies.	Promising outcomes in selected trials; effect sizes vary by indication and methodology.
**Advantages**	Defined agents; targeted modulation; favorable safety in most settings.	Low cost; scalable; leverages endogenous microbiota; diet‐compatible.	Largest ecological reset; potential for robust clinical changes when engraftment succeeds.
**Main limitations**	Strain/product variability; heterogeneous outcomes; delivery/stability challenges; need for indication–strain matching.	Response depends on baseline microbiota/diet; heterogeneous readouts; standardization of substrates/doses needed.	Donor screening and standardization; safety/logistics; methodological heterogeneity and small samples.
**Standardization & scalability**	Relatively high (well‐defined strains) but requires harmonized outcomes and delivery.	High (defined substrates) but requires harmonized protocols/outcomes.	Lower; rigorous donor selection and harmonized protocols needed; scalability challenging.
**Overall positioning**	More defined and generally safer; typically, subtler effects; often strain‐specific.	Indirect, host‐dependent modulation; complementary to probiotics.	Broad ecosystem reorganization with potentially larger but less predictable effects.

## 4 Evidence of Probiotic Use in Specific Neurological Disorders (Table 2)

### 4.1 Depression and Anxiety Disorders

Depression and anxiety are among the most prevalent mental health disorders, characterized by persistent low mood, anhedonia, excessive worry, and various somatic symptoms with‐out clear physiological causes [39]. Globally, depression affects more than 350 million people, contributing to substantial disability and socioeconomic burden and underscoring the urgent need for innovative and accessible therapeutic strategies [40]. While conventional treatments, including pharmacotherapy and psychotherapy, remain the cornerstone of management, growing interest in the GBA has prompted exploration of microbiota‐targeted interventions. Probiotics have emerged as a promising adjunctive approach due to their ability to modulate gut microbiota composition, reduce systemic inflammation, and influence neurotransmitter synthesis involved in mood regulation [41].

**Table 2:** Conceptual distinctions between probiotics, psychobiotics, synbiotics, and postbiotics.

Term	Definition	Examples/Components	Mechanism of action/Neurological relevance	Limitations/Considerations	Refs
**Probiotics**	Live microorganisms that confer health benefits when administered in adequate amounts (WHO/FAO definition)	Lactobacillus rhamnosus, Bifidobacterium bifidum	Modulate gut–brain axis via immune regulation, SCFAs, neurotransmitter modulation	Strain‐specific effects; variability across hosts	[30, 38]
**Psychobiotics**	Subclass of probiotics with specific effects on mental health and cognition	L. rhamnosus (GABA modulation), B. longum (stress resilience)	Produce neuroactive metabolites (GABA, serotonin, dopamine); regulate HPA axis and stress response	Still emerging; limited large‐scale RCTs; heterogeneous definitions	[7].
**Synbiotics**	Combination of probiotics and prebiotics providing synergistic health benefits	Probiotic blend + inulin, FOS, GOS	Enhance colonization, improve SCFA production, synergistic modulation of gut microbiota and immune pathways	Optimal formulations not standardized; variable outcomes	[13]
**Postbiotics**	Nonviable microbial products, metabolites, or cell‐wall components that confer health benefits	SCFAs (butyrate, propionate), bacteriocins, exopolysaccharides	Anti‐inflammatory, strengthen barrier integrity, potential neuroprotection through immune/metabolic modulation	Regulatory definitions evolving; few clinical data	[24]

However, probiotics offer distinct advantages including a favorable safety profile with minimal adverse effects and may be particularly valuable for patients who are reluctant to use or unable to tolerate conventional medications [42, 43]. Most importantly, randomized controlled trials suggest that probiotics may serve as effective adjunctive therapies, enhancing the efficacy of antidepressants in reducing treatment‐resistant symptoms and improving quality of life. Therefore, rather than positioning probiotics as substitutes for established psychiatric medications, the evidence supports their role as low‐risk complementary strategies that may optimize treatment outcomes when integrated with standard care, particularly in patients seeking multimodal therapeutic approaches or experiencing suboptimal responses to conventional treatments alone [44, 45].

Alterations in gut microbiota, particularly reductions in Firmicutes and increases in Bacteroidetes and Proteobacteria, have been associated with depressive symptoms. Probiotic supplementation, particularly with strains of Lactobacillus and Bifidobacterium, has shown beneficial effects in some clinical trials. However, findings regarding anxiety are more variable, likely due to differences in study design, probiotic strains, and sample sizes [39]. Although still in its early stages, research into psychobiotics offers a novel and biologically plausible approach to managing mood disorders. Continued clinical investigation is needed to clarify strain‐specific effects and optimize therapeutic regimens.

### 4.2 Alzheimer's Disease and Cognitive Decline

Alzheimer's disease (AD), the most common form of dementia, is characterized by progressive cognitive decline and neurodegeneration. Emerging evidence suggests a strong link between gut microbiota and AD pathogenesis, particularly through neuroinflammation, oxidative stress, and disruption of the gut–brain axis [46]. Animal studies have shown that probiotic supplementation can reduce amyloid‐beta (Aβ) accumulation and improve cognitive function by modulating inflammatory responses and enhancing antioxidant activity. For example, in transgenic AD mouse models, probiotics such as *Lactobacillus plantarum* and *Bifidobacterium breve* have improved memory performance and reduced hippocampal inflammation [47].

Human studies, although still limited, support these findings. A double‐blind randomized controlled trial (RCT) by Akbari et al. in a small sample (*n* = 60) showed that 12 weeks of multistrain probiotic milk supplementation resulted in significant improvements in Mini‐Mental State Examination (MMSE) scores in Alzheimer's patients compared to a milk control group; since microbiota assessment (fecal markers) was not performed and only a cognitive test was used, the sustainability of the effects in more diverse cognitive tests or over longer periods remains uncertain [48]. Improvements were accompanied by reductions in serum malondialdehyde and high‐sensitivity C‐reactive protein, suggesting decreased oxidative stress and systemic inflammation.

While promising, most clinical studies are small and short‐term. Thus, further large‐scale RCTs are necessary to confirm the long‐term efficacy and identify optimal probiotic strains and dosages for AD prevention or treatment [49].

### 4.3 Parkinson's Disease

Parkinson's disease (PD) is increasingly associated with gut dysbiosis, with gastrointestinal (GI) disturbances—particularly constipation—often preceding motor symptoms by several years. Alterations in the gut microbiota in PD include reduced populations of butyrate‐producing bacteria (e.g., *Roseburia* spp.) and increased abundance of pro‐inflammatory taxa (e.g., *Enterobacteriaceae*), contributing to intestinal permeability (“leaky gut”), systemic inflammation, and neurodegenerative processes [50]. These changes may facilitate α‐synuclein aggregation, a hallmark of PD pathology, through gut–brain axis disruption. Probiotics have shown promise as adjunctive therapies in managing PD‐related symptoms. Strains such as *Lactobacillus casei*, *Bifidobacterium longum*, and *Clostridium butyricum* have demonstrated beneficial effects by improving gut motility, reinforcing gut barrier integrity, reducing endotoxin translocation, and lowering circulating pro‐inflammatory cytokines like TNF‐α and IL‐6 [50].

In preclinical studies, *C. butyricum* enhanced intestinal function and increased the production of neuroprotective SCFAs such as butyrate. Clinical trials using multi‐strain formulations (*Lactobacillus*/*Bifidobacterium* blends) have also reported improvements in constipation and modest alleviation of motor symptoms in PD patients [51]. Currently available evidence supports symptom relief, particularly for gastrointestinal complaints such as constipation, with secondary improvements in quality of life; in contrast, evidence for direct effects on motor symptoms or disease progression is scarce and limited by small, short‐term studies or heterogeneous trial designs. Further research is needed to identify specific probiotic formulations for Parkinson's and validate their long‐term efficacy through well‐designed randomized controlled trials [23, 50, 51].

### 4.4 Autism Spectrum Disorder

Alterations in gut microbiota composition have been consistently observed in individuals with ASD and are associated with both GI symptoms and neuroinflammatory processes. Dysbiosis involving genera such as *Bifidobacterium*, *Lactobacillus*, and *Clostridium* may disrupt neurotransmitter synthesis and immune regulation, potentially contributing to ASD pathophysiology [52]. Probiotic interventions targeting the gut–brain axis have emerged as promising adjunctive strategies. Mechanistic studies suggest that probiotics may ameliorate ASD‐related symptoms by modulating inflammatory cytokine levels, reducing neuroinflammation, and promoting the synthesis of neurotransmitters such as GABA and dopamine [53].

Rojo‐Martísella et al. demonstrated that supplementation with *Lactiplantibacillus plantarum* and *Levylactobacillus brevis* improved impulsivity and quality of life in children with ASD [52]. Similarly, a randomized controlled trial by Khanna et al. reported that a 3‐month probiotic intervention significantly improved behavioral symptoms, including social withdrawal and hyperactivity, as well as GI complaints in children with ASD [54]. This trial suggests a significant correlation between the severity of behavioral and GI symptoms in children with ASD, such that the available evidence suggests that subgroups of children with prominent GI symptoms may be more responsive to probiotics; However, the certainty is not yet high. Consequently, response to treatment may differ in subgroups of children with ASD [54, 55, 56].

Despite these encouraging findings, results across studies remain heterogeneous. Meta‐analyses emphasize the need for more rigorous and large‐scale trials to confirm efficacy and identify responsive subpopulations [57]. Nevertheless, current evidence supports the use of probiotics as a complementary therapeutic approach, particularly in ASD patients with prominent GI dysfunction.

### 4.5 Multiple Sclerosis and Other Neuroinflammatory Conditions

Neuroinflammation plays a pivotal role in the progression of neurodegenerative and neuropsychiatric disorders, including MS. MS is a chronic autoimmune condition characterized by demyelination and CNS inflammation. Growing evidence suggests that gut dysbiosis contributes to the immunopathogenesis of MS through dysregulation of the gut–immune–brain axis. Probiotic administration has shown potential in modulating this axis. Strains such as *Lactobacillus plantarum*, *Bifidobacterium lactis*, and *Streptococcus thermophilus* have been found to shift immune responses by downregulating Th1/Th17 activity, enhancing regulatory T‐cell differentiation, and reducing the secretion of pro‐inflammatory cytokines including IL‐17 and IFN‐γ [58].

Both clinical and preclinical studies, including experimental autoimmune encephalomyelitis (EAE) models, have reported symptom alleviation and reduced disease severity following probiotic supplementation, with genera like *Prevotella* implicated in beneficial outcomes [59]. Additionally, probiotics may counteract age‐related gut dysbiosis and increased intestinal permeability, thereby mitigating systemic and CNS inflammation [60]. A murine study by Pei et al. showed that extracellular vesicles derived from probiotic‐modified gut microbiota could cross the blood–brain barrier and suppress hippocampal inflammation, resulting in measurable behavioral improvements [61]. These findings support the therapeutic potential of probiotics in restoring immune homeostasis and reducing chronic neuroinflammation in MS and other neuroinflammatory conditions.

Since the findings in MS come from animal models of EAE, which provide mechanistic insights, they may not fully reflect the complexity of human disease due to differences in immune regulation, microbiota composition, and disease heterogeneity between rodents and humans [58, 61]. Although further clinical validation and larger human trials are needed, probiotics are a promising adjunct to conventional therapies in such disorders (Figure 2).

## 5 Safety, Limitations, and Considerations

Probiotics are generally regarded as safe for most individuals, with adverse events typically limited to mild gastrointestinal symptoms such as bloating, gas, or diarrhea. However, there is evidence of specific gastrointestinal side effects associated with probiotic use, such as small intestinal bacterial overgrowth (SIBO) with brain fog or rare but serious systemic infections such as Lactobacillus bacteremia and Saccharomyces fungemia [38]. Patients taking immunosuppressive drugs and chemotherapy, those with neutropenia, active colitis/risk of perforation, valvular heart disease, history of endocarditis, transplant recipients, autoimmune diseases, and individuals with central venous catheters are at‐risk populations. In addition to the potential benefits of probiotics for preterm infants, careful adherence to quality control and screening for antimicrobial resistance (AMR) genes is essential [62].

A major limitation in the current literature is the strain‐specific nature of probiotic effects. Additionally, concerns have been raised about antibiotic resistance gene transfer and the potential for probiotics to disrupt the native microbiota, with some evidence suggesting delayed microbiome recovery when co‐administered with antibiotics. While regulatory screening minimizes these risks, long‐term safety data are limited, emphasizing the need for careful monitoring in future large‐scale trials. Interindividual variability in gut microbiota composition further contributes to inconsistent responses [30].

Interindividual variability in gut microbiota composition further contributes to inconsistent responses, making it difficult to predict treatment outcomes without personalized profiling. Many existing studies are limited by small cohorts, short intervention durations, and inconsistent methodologies. These factors hinder the ability to draw robust, reproducible conclusions. Moreover, the long‐term safety and efficacy of probiotic use in neurologic populations—particularly in the elderly or those with advanced neurodegenerative diseases—remain uncertain [30]. Despite these challenges, probiotics offer a low‐risk, potentially beneficial adjunct to conventional therapies. Future investigations should focus on large‐scale, standardized clinical trials to determine optimal strains, dosages, and treatment durations, while exploring personalized approaches based on host microbiome characteristics [38].

## 6 Future Directions and Perspectives

The therapeutic potential of probiotics in neurological disorders is a rapidly evolving field, particularly within the framework of personalized medicine. Advances in microbiome profiling may enable the development of individualized probiotic interventions tailored to a patient's unique gut microbial composition. Although such personalized approaches could increase treatment efficacy, reduce variability in outcomes, and minimize side effects [63]. This approach is currently only feasible on a limited scale and based on simple clinical phenotypes (e.g., bowel habit patterns) [64]. However, its widespread application is not yet realistic due to the high cost and time of microbiome assessments, the high interindividual variability of the microbiota, and the insufficient standardization of laboratory procedures and strain/product safety requirements [64, 65].

Emerging evidence suggests that personalized biomarkers could help predict who will benefit most from probiotic interventions. Baseline microbiota diversity, higher levels of Lactobacillus and Bifidobacterium, and lower abundance of pro‐inflammatory taxa appear linked to better outcomes in neurological and psychiatric disorders [66, 67]. Similarly, reductions in inflammatory markers such as IL‐6, TNF‐α, IL‐17, and CRP, along with favorable metabolomic profiles like elevated SCFAs and balanced tryptophan metabolism, correlate with improved cognition and mood [68, 69]. Host–microbe interaction measures, including gut permeability and vagal tone, may further refine responder prediction. Integrating multi‐omics approaches into clinical trials will be essential for developing biomarker‐guided, personalized probiotic therapies [67, 70, 71].

Future research should prioritize large‐scale, randomized controlled trials (RCTs) that evaluate the long‐term cognitive, emotional, and neurological outcomes of probiotic supplementation. In addition, emerging strategies such as synbiotics, combinations of probiotics and prebiotics, and postbiotics, bioactive metabolites produced by probiotics, offer promising therapeutic avenues that may enhance host–microbiota interactions and yield greater clinical benefits [72].

In addition, strain standardization remains a critical step toward generating robust clinical evidence. The heterogeneity of current probiotic formulations—including variability in strains, dosages, viability, and delivery systems—limits reproducibility and complicates direct comparisons across studies [30, 73]. Future research should prioritize genomic characterization, standardized production processes, dose–response evaluations, and mechanistic validation of strain‐specific neurobiological effects. Only with these foundations can large‐scale randomized controlled trials be reliably conducted to establish both the safety and clinical efficacy of probiotics.

Although limited, evidence has suggested the effect of postbiotics on reducing inflammatory biomarkers and strengthening the intestinal epithelial barrier with a greater safety and stability advantage than synbiotics [74, 75]. Moreover, synbiotics with the synergistic ability of probiotics and prebiotics can have an effect on reducing inflammatory biomarkers and hold promise in personalized chronic interventions [76]. Integrating probiotics with standard pharmacological and behavioral treatments may lead to a more holistic and multimodal approach to managing neurodegenerative and psychiatric conditions. By simultaneously addressing microbial dysbiosis, neuroinflammation, and neurotransmitter imbalances, probiotics may eventually play a central role in both the prevention and treatment of neurological disorders [77].

## 7 Conclusions

The gut–brain axis represents a promising target for addressing neurological and neuropsychiatric disorders. Probiotics have shown potential to reduce neuroinflammation, restore microbial balance, and modulate neurotransmitter pathways, with encouraging evidence in depression, anxiety, Parkinson's disease, Alzheimer's disease, ASD, and MS.

Nevertheless, the field faces substantial limitations. Current studies are often small, heterogeneous, and strain‐specific, with inconsistent outcomes across populations. A major barrier to clinical translation is the absence of standardized probiotic formulations, agreed dosing regimens, and long‐term safety data. These gaps limit reproducibility and hinder regulatory approval, making widespread clinical adoption premature. Future research should focus on large, well‐controlled trials, standardization of probiotic preparations, and integration of multi‐omics approaches to identify biomarkers of response. Efforts to personalize probiotic interventions and explore synergistic combinations with diet or pharmacological therapies will also be essential.

## Author Contributions

Mahla Jafari and Meysam Hasannejad‐Bibalan contributed to the study's conception and design. Morteza Alipour, Sara Zamani, Aryan Mohtasham Amiri, and Parsin Pourabbas conducted comprehensive search on literatures. Mahla Jafari, Morteza Alipour, Sara Zamani, Aryan Mohtasham Amiri, Parsin Pourabbas, Meysam Hasannejad‐Bibalan commented evaluated studies and wrote the first draft. All authors read and approved the final manuscript.

## Ethics Statement

The authors have nothing to report.

## Consent

The authors obtained consent to publish. The current manuscript contains no individual person's data. Therefore, consent to publish is not applicable.

## Conflicts of Interest

The authors declare no conflicts of interest.

## Transparency Statement

The lead author Meysam Hasannejad‐Bibalan affirms that this manuscript is an honest, accurate, and transparent account of the study being reported; that no important aspects of the study have been omitted; and that any discrepancies from the study as planned (and, if relevant, registered) have been explained.

## Data Availability

The data that support the findings of this study are available from the corresponding author upon reasonable request. All authors have read and approved the final version of the manuscript. The corresponding author, Meysam Hasannejad‐Bibalan takes complete responsibility for the integrity of the data.
